# Supplementary material for: Genome-Wide Identification of the CDPK Gene Family and Their Involvement in Taproot Cracking in Radish
Source: Int J Mol Sci. 2023 Oct 11;24(20):15059. doi: 10.3390/ijms242015059 (PMC10606364; doi:10.3390/ijms242015059)
Supplement: Supplementary file 1 [file ijms-24-15059-s001.zip › ijms-2599138-supplementary.pdf]

Supplementary Data

Genome-Wide Identification of the CDPK Gene Family and Their Involvement in Taproot Cracking in Radish

Qian Yang †, Yan Huang †, Lei Cui, Caixia Gan, Zhengming Qiu, Chenghuan Yan * and Xiaohui Deng *

Key Laboratory of Vegetable Ecological Cultivation on Highland, Ministry of Agriculture and Rural Affairs, Hubei Key Laboratory of Vegetable Germplasm Innovation and Genetic Improvement, Institute of Economic Crops, Hubei Academy of Agricultural Sciences, Wuhan 430070, China; yangqian@hbaas.com (Q.Y.); huangyanzi@hbaas.com (Y.H.); cuilei18062099715@hbaas.com (L.C.); gancaixia@hbaas.com (C.G.); qiusunmoon@hbaas.ac.cn (Z.Q.)

* Correspondence: yanch@hbaas.ac.cn (C.Y.); dengshine@hbaas.com (X.D.)

† These authors contributed equally to this work.

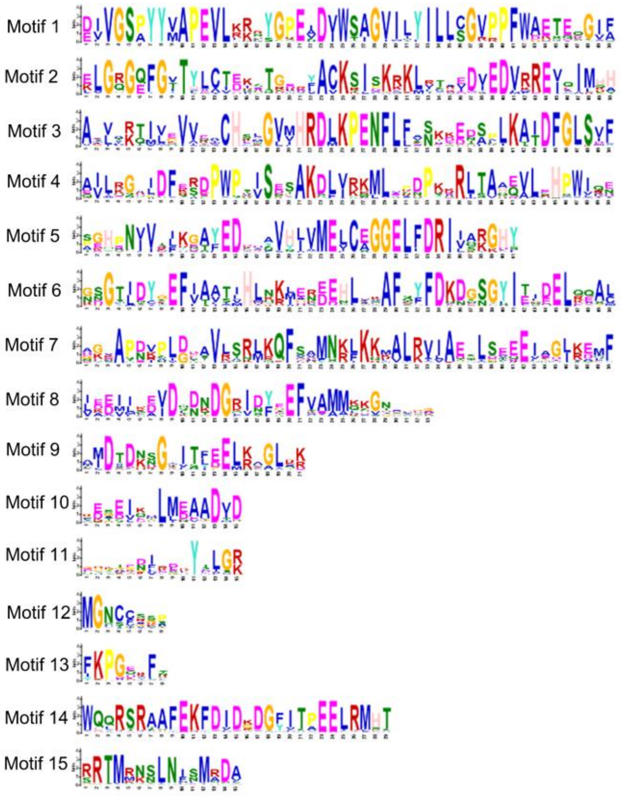

Figure S1. Conserved motifs of *RsCDPK* protein sequences.

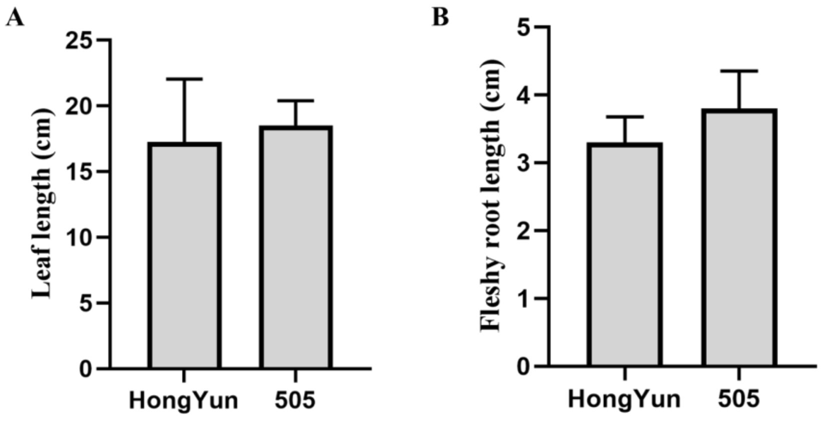

Figure S2. The length statistics of "HongYun" and "505" in of **(A)** leaf and **(B)** fleshy root. All values represent the means ± SD of 3 independent experiments.

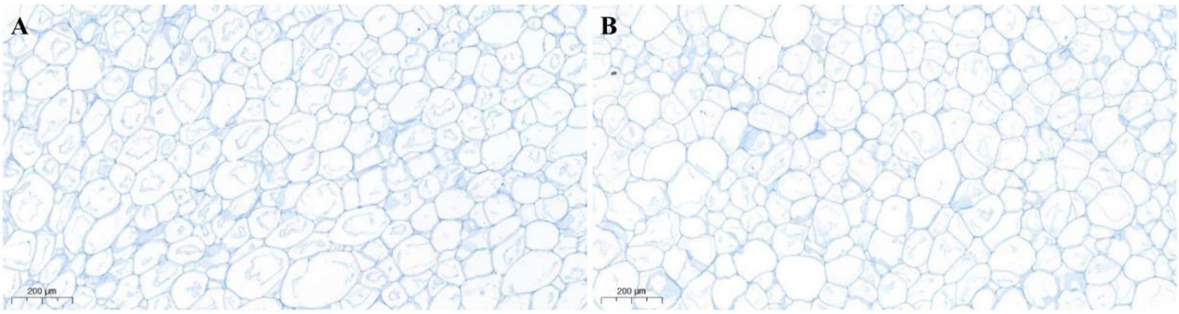

Figure S3. Cytological observation taproot flesh cells of **(A)** "HongYun" and **(B)** "505." Scale bar = 200 μm.

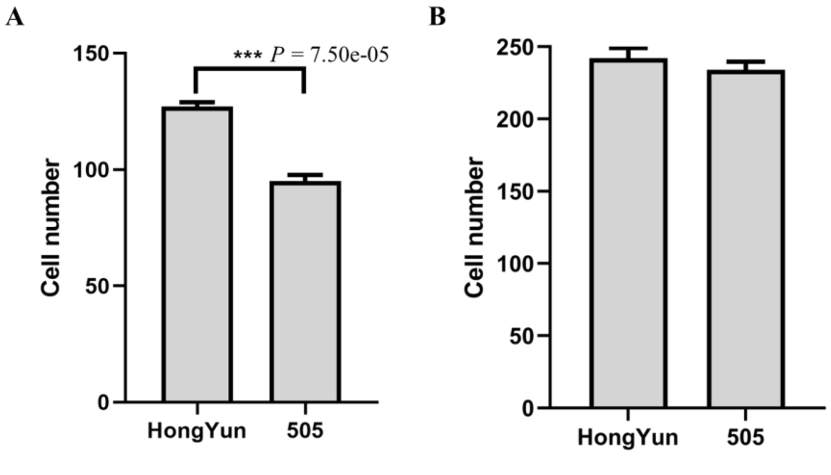

Figure S4. Cell number statistics of "HongYun" and "505" in of **(A)** taproot cortex cells (50 μm vision) and **(B)** taproot flesh cells (200 μm vision). All values are the means ± SD of 3 independent experiments. Statistically significant differences (Student's *t*-test) are indicated as follows: **, $P < 0.01$.

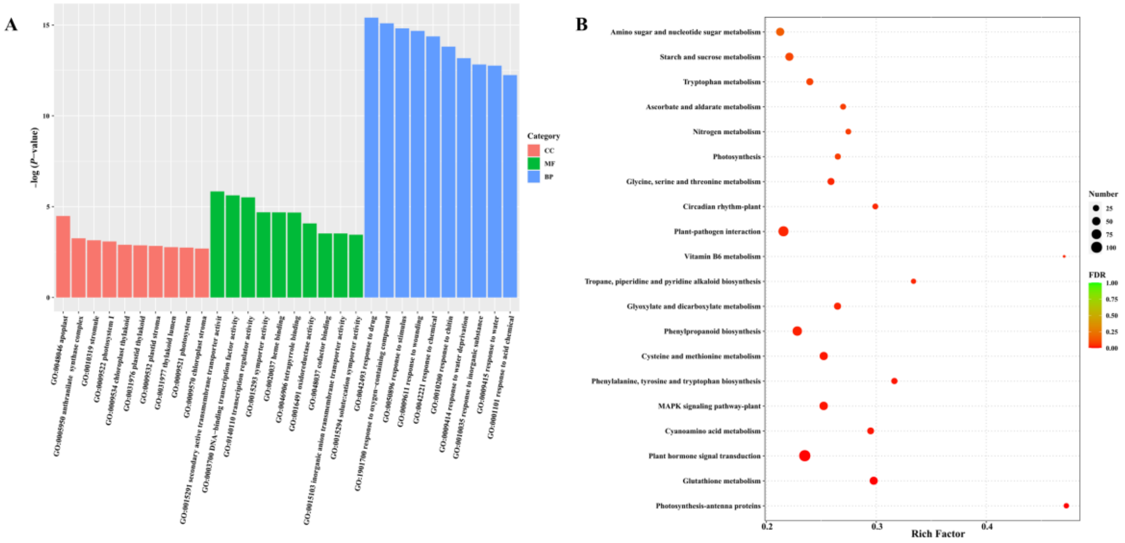

Figure S5. Gene Ontology (GO) and Kyoto Encyclopedia of Genes and Genomes (KEGG) analyses of differentially expressed genes. Enriched (**A**) GO terms and (**B**) KEGG pathways. *P*-value < 0.05.

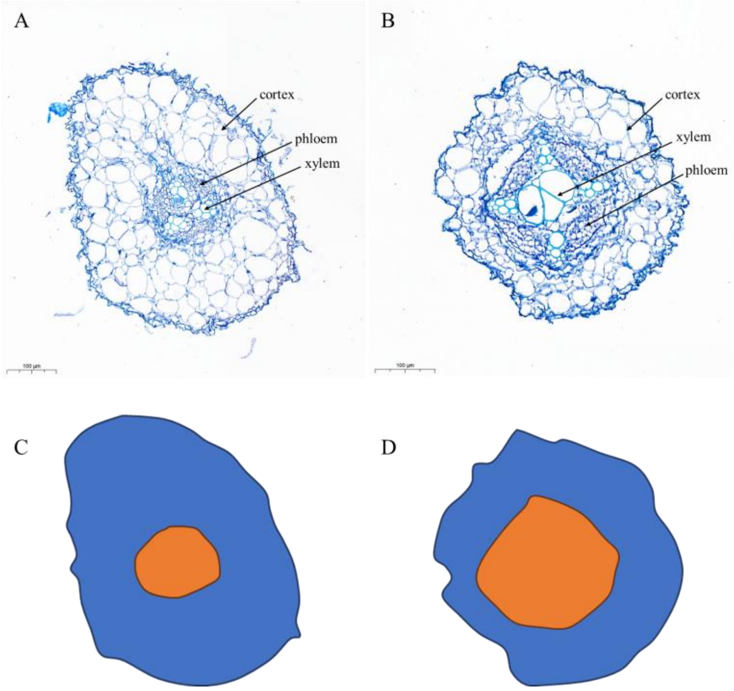

Figure S6. Cytological observation and contour projection of the transgenic roots of (A, C) EV and (B, D) RsCDPK21OE. Blue represents the cross-sectional area of the root, and orange represents the xylem and phloem regions. Scale bar = 100 μm.

Table S1. Identification and analysis of putative RsCDPK genes in radish.

Gene name	Gene ID	Start	End	5′~3′	Chromosome	Protein length (aa)	pI	Molecular weight	Localization
RsCDPK1	*R120188160*	6,230,472	6,233,000	+	Rs7	607	5.31	67,471.90	Nucleus
RsCDPK2	*R120091160*	42,315,753	42,318,128	+	Rs5	634	5.36	70,184.67	Nucleus
RsCDPK3	*R120037900*	37,246,094	37,248,420	-	Rs2	524	6.06	58,695.72	Nucleus
RsCDPK4	*R120051840*	44,716,270	44,718,388	-	Rs4	437	7.19	49,123.61	Nucleus
RsCDPK5	*R120046610*	42,007,984	42,010,167	+	Rs2	555	5.54	62,140.48	Nucleus
RsCDPK6	*R120206190*	34,260,471	34,262,777	+	Rs9	549	5.19	61,554.83	Nucleus
RsCDPK7	*R120075560*	7,426,735	7,428,876	-	Rs4	523	5.81	58,645.32	Nucleus
RsCDPK8	*R120351210*	23,396,805	23,399,159	-	Rs2	536	5.85	60,166.64	Nucleus
RsCDPK9	*R120303360*	5,922,293	5,924,580	+	Rs6	539	5.93	59,920.89	Nucleus
RsCDPK10	*R120284520*	8,759,026	8,760,942	-	Rs1	503	6.14	56,306.67	Nucleus
RsCDPK11	*R120119790*	24,093,410	24,095,496	+	Rs5	498	5.17	56,126.19	Nucleus
RsCDPK12	*R120156070*	11,946,955	11,949,262	-	Rs6	491	5.21	55,713.12	Nucleus
RsCDPK13	*R120340110*	48,060,636	48,063,669	+	Rs4	486	5.99	54,472.52	Nucleus
RsCDPK14	*R120079840*	4,538,320	4,540,367	+	Rs4	471	6.51	52,178.19	Nucleus
RsCDPK15	*R120085600*	1,250,768	1,253,085	-	Rs4	517	5.5	57,666.22	Nucleus
RsCDPK16	*R120100730*	36,859,838	36,862,710	-	Rs5	574	9.35	64,884.98	Nucleus
RsCDPK17	*R120094970*	40,188,686	40,191,003	-	Rs5	538	5.59	60,116.22	Nucleus
RsCDPK18	*R120085980*	1,046,617	1,049,215	+	Rs4	542	6.28	61,481.03	Nucleus
RsCDPK19	*R120101030*	36,683,835	36,686,110	+	Rs5	550	5.23	61,621.72	Nucleus
RsCDPK20	*R120227860*	23,980,818	23,983,358	+	Rs3	607	5.43	67,298.62	Nucleus
RsCDPK21	*R120123230*	38,068,019	38,070,103	-	Rs6	519	5.88	58,578.59	Nucleus
RsCDPK22	*R120141160*	52,434,928	52,437,272	+	Rs6	597	6.71	67,730.44	Nucleus
RsCDPK23	*R120167160*	23,494,913	23,497,779	+	Rs6	575	9.34	64,987.18	Nucleus
RsCDPK24	*R120057990*	40,592,418	40,595,025	-	Rs4	582	6.01	66,212.62	Nucleus
RsCDPK25	*R120167750*	24,408,065	24,410,329	-	Rs6	513	5.23	57,379.09	Nucleus
RsCDPK26	*R120193080*	3,755,960	3,758,614	+	Rs7	498	5.53	55,541.27	Nucleus
RsCDPK27	*R120197410*	1,291,482	1,293,651	+	Rs7	472	5.85	52,900.34	Nucleus

RsCDPK28	*R120138420*	50,483,538	50,486,367	+	Rs6	532	8.75	60,129.33	Nucleus
RsCDPK29	*R120184750*	8,346,464	8,349,522	+	Rs7	529	5.52	59,687.88	Nucleus
RsCDPK30	*R120185690*	7,759,367	7,761,294	-	Rs7	499	6.02	56,278.57	Nucleus
RsCDPK31	*R120173700*	16,761,538	16,763,933	+	Rs7	539	5.89	61,085.47	Nucleus
RsCDPK32	*R120221890*	45,207,665	45,210,531	+	Rs9	544	8.99	60,996.17	Nucleus
RsCDPK33	*R120351270*	23,344,038	23,346,110	-	Rs2	518	5.56	57,678.47	Nucleus
RsCDPK34	*R120231700*	26,898,508	26,900,802	-	Rs3	541	7.98	61,304.20	Nucleus
RsCDPK35	*R120244530*	34,751,794	34,754,078	+	Rs3	584	5.26	65,274.06	Nucleus
RsCDPK36	*R120310420*	4,123,653	4,127,223	-	Rs3	550	5.58	62,421.51	Nucleus
RsCDPK37	*R120320250*	17,693,066	17,695,460	+	Rs8	354	5.05	39,777.12	Nucleus

Table S2. Gene pairs of RsCDPK for segmental duplication.

Gene name	Gene name
RsCDPK3	*RsCDPK14*
RsCDPK5	*RsCDPK15*
RsCDPK8	*RsCDPK27*
RsCDPK10	*RsCDPK21*
RsCDPK10	*RsCDPK30*
RsCDPK16	*RsCDPK23*
RsCDPK16	*RsCDPK32*
RsCDPK17	*RsCDPK9*
RsCDPK19	*RsCDPK25*
RsCDPK24	*RsCDPK22*
RsCDPK25	*RsCDPK6*
RsCDPK34	*RsCDPK22*
RsCDPK35	*RsCDPK1*

Table S3. Conserved motif distributions of RsCDPK members.

Group	Genes ID	Motifs
I	RsCDPK25	12, 11, 2, 5, 1, 4, 7, 9, 10, 6, 8, 15
I	RsCDPK6	12, 11, 2, 5, 3, 13, 1, 4, 7, 9, 10, 6, 8, 15
I	RsCDPK19	12, 11, 2, 5, 3, 13, 1, 4, 7, 9, 10, 6, 8, 15
I	RsCDPK5	12, 11, 2, 5, 3, 13, 1, 4, 7, 9, 10, 6, 8, 15
I	RsCDPK15	12, 11, 2, 5, 1, 4, 7, 9, 10, 6, 8, 15
I	RsCDPK36	11, 2, 5, 3, 13, 1, 4, 7, 9, 10, 6, 8, 15
I	RsCDPK11	11, 2, 5, 3, 13, 1, 4, 7, 9, 10, 6, 8, 15
I	RsCDPK37	11, 2, 5, 3, 13, 1, 9, 8, 15
I	RsCDPK12	11, 2, 5, 3, 13, 1, 4, 7, 9, 10, 6, 8, 15
I	RsCDPK20	12, 11, 2, 5, 3, 13, 1, 4, 7, 9, 10, 6, 8
I	RsCDPK2	12, 11, 2, 5, 3, 13, 1, 4, 7, 9, 10, 6, 8
I	RsCDPK1	12, 11, 2, 5, 3, 13, 1, 4, 7, 9, 10, 6, 8
I	RsCDPK35	11, 2, 5, 3, 13, 1, 4, 7, 9, 10, 6, 8
II	RsCDPK3	11, 2, 5, 3, 13, 1, 4, 7, 9, 10, 6, 8
II	RsCDPK14	11, 2, 5, 3, 13, 1, 4, 7, 9, 10
II	RsCDPK33	12, 11, 2, 5, 3, 13, 1, 4, 7, 9, 10, 6, 8
II	RsCDPK29	12, 11, 2, 5, 3, 13, 1, 4, 9, 10, 6, 8
II	RsCDPK9	12, 11, 2, 5, 3, 13, 1, 4, 7, 9, 10, 6, 8
II	RsCDPK17	12, 11, 2, 5, 3, 13, 1, 4, 9, 10, 6, 8
III	RsCDPK34	12, 11, 2, 3, 13, 1, 4, 7, 9, 10, 6, 8
III	RsCDPK22	12, 11, 2, 5, 3, 13, 1, 4, 7, 9, 10, 6, 8
III	RsCDPK24	12, 11, 2, 5, 3, 13, 1, 4, 7, 9, 10, 6, 8
III	RsCDPK27	12, 11, 2, 5, 3, 13, 1, 4, 7, 9, 10, 6
III	RsCDPK8	12, 11, 2, 5, 3, 13, 1, 4, 7, 9, 10, 6, 8
III	RsCDPK26	12, 11, 2, 5, 3, 13, 1, 4, 7, 9, 10, 6, 10

III	RsCDPK4	12, 11, 2, 3, 1, 4, 7, 9, 10, 6
III	RsCDPK31	12, 11, 2, 5, 3, 13, 1, 4, 7, 9, 10, 6, 8
III	RsCDPK13	12, 11, 2, 5, 3, 13, 1, 4, 7, 9, 10, 6
III	RsCDPK7	12, 11, 2, 5, 3, 13, 1, 4, 7, 9, 10, 6
III	RsCDPK30	12, 11, 2, 5, 3, 13, 1, 4, 7, 9, 10, 6, 10
III	RsCDPK10	12, 12, 11, 2, 5, 3, 13, 1, 4, 7, 9, 10, 6
III	RsCDPK21	12, 11, 2, 5, 3, 13, 1, 4, 7, 9, 10, 6
IV	RsCDPK28	12, 11, 2, 5, 3, 13, 1, 4, 7, 9, 8, 14, 8
IV	RsCDPK32	12, 11, 2, 5, 3, 13, 1, 4, 7, 9, 8, 14, 8
IV	RsCDPK18	12, 11, 2, 5, 3, 13, 1, 4, 7, 9, 8, 14, 8
IV	RsCDPK16	12, 11, 2, 5, 3, 13, 1, 4, 7, 9, 8, 14, 8
IV	RsCDPK23	12, 11, 2, 5, 3, 13, 1, 4, 7, 9, 8, 14, 8

Table S4. Sequencing and mapping of RNA-seq clean data.

Sample	replicate	Clean Data (bp)	Clean_Reads	Q30 (%)	N (%)	Total_Mapped	Uniquely_Mapped	Multiple_Mapped
N-Cracking	HongYun_1	5,630,328,900	37,535,526	91.11	0.000811	29,781,004 (79.34%)	28,685,612 (96.32%)	1,095,392 (3.68%)
	HongYun_2	5,478,037,500	36,520,250	91.34	0.000809	29,044,114 (79.53%)	28,017,261 (96.46%)	1,026,853 (3.54%)
	HongYun_3	5,130,747,300	34,204,982	90.64	0.000813	27,183,371 (79.47%)	26,222,610 (96.47%)	960,761 (3.53%)
Cracking	505_1	5,264,891,400	35,099,276	91.29	0.000807	27,769,383 (79.12%)	26,709,228 (96.18%)	1,060,155 (3.82%)
	505_2	6,970,401,900	46,469,346	90.51	0.000811	36,843,549 (79.29%)	35,425,558 (96.15%)	1,417,991 (3.85%)
	505_3	5,925,855,300	39,505,702	90.27	0.000803	31,153,019 (78.86%)	29,950,091 (96.14%)	1,202,928 (3.86%)

Table S5. Data analysis of genes expression value.

Gene ID	Non-cracking		Cracking		$LSD_{0.05}$	Data
	Mean	S.D.	Mean	S.D.		
RsCDPK22	0.0521	0.0024	1.4348	0.2928	0.724	RNA-seq
RsCDPK33	1.0413	0.9357	5.3537	1.1290	0.481	RNA-seq
RsCDPK12	1.8494	0.3627	14.5896	2.3539	5.267	RNA-seq
RsCDPK6	4.5459	1.8827	26.2673	8.0595	16.683	RNA-seq
RsCDPK10	2.9962	0.8415	16.7553	4.4066	9.045	RNA-seq
RsCDPK21	41.3175	3.5658	85.7007	4.9518	15.185	RNA-seq
RsCDPK22	0.0147	0.0089	0.0723	0.0149	0.028	RT-qPCR
RsCDPK33	0.0071	0.0048	0.0562	0.0113	0.027	RT-qPCR
RsCDPK12	0.0580	0.0310	1.5688	0.2631	0.655	RT-qPCR
RsCDPK6	0.3224	0.0440	2.0996	0.2267	0.0668	RT-qPCR
RsCDPK10	0.5260	0.1909	2.0900	0.3441	1.041	RT-qPCR
RsCDPK21	2.4064	0.2742	7.6349	1.7171	4.392	RT-qPCR

Table S6. Data analysis of *RsCDPK21* genes expression value.

Gene ID	EV		RsCDPK21OE		$LSD_{0.05}$
	Mean	S.D.	Mean	S.D.	
RsCDPK21	0.0015	0.0003	0.4301	0.0516	0.128

Table S7. Data analysis of Root length.

Length (mm)	EV		RsCDPK21OE		$LSD_{0.05}$
	Mean	S.D.	Mean	S.D.	
Root	99.6	3.0496	169.1	10.977	8.75

Table S8. Primers used in this study.

Primer name	Primer sequences (5′-3′)	Purpose
RsCDPK22-F	GGTCACTTGACTTTTGAGGAAC	RT-qPCR
RsCDPK22-R	CAGTGTCACAAACTCTTCACAG	
RsCDPK33-F	GCTCAAGCAATTCAAAGCAATG	RT-qPCR
RsCDPK33-R	TCATCGGTGTCCATTCCTTTAA	
RsCDPK12-F	AAGGACAGTATGGAACCACTTC	RT-qPCR
RsCDPK12-R	GCATTATCTGGATCTCCCTCAA	
RsCDPK6-F	TTCTCGGTCACAAGACTCCT	RT-qPCR
RsCDPK6-R	ACCAGCCAAATGGTGCATTA	
RsCDPK10-F	GAAGATCACCGACAAGTACACC	RT-qPCR
RsCDPK10-R	GTAAGTCTCCCTGAGCTTGAC	
RsCDPK21-F	CGGCATCAAGGACGTGATAA	RT-qPCR
RsCDPK21-R	GCAGAGTGGACATGATCGAG	
Rs.RPII-F	ACAGGTTGTGGTGGTCGATC	RT-qPCR
Rs.RPII-R	CCTTCACTGCACCTGTGGAT	
GmELF1b-F	GTTGAAAAGCCAGGGGACA	RT-qPCR
GmELF1b-R	TCTTACCCCTTGAGCGTGG	
35SRsCDPK21-F	gtcgactctagaggatccATGGGTAACTGTAACGTCTG	Vector construction
35SRsCDPK21-R	actcatttttctaccggtaccgtTTAGAGATTAGACAGAACCTTG	
